# ROLE ENGAGEMENT IN VOLUNTEERING: IMPACTS ON SUBJECTIVE WELL-BEING AND COGNITION IN MIDDLE AND LATER LIFE

**DOI:** 10.1093/geroni/igae098.3707

**Published:** 2024-12-31

**Authors:** Shiyu Lu

**Affiliations:** The University of Hong Kong, Hong Kong, China (People’s Republic)

## Abstract

The existing literature predominantly emphasizes the quantitative aspect of volunteering, such as volunteer hours, overlooking the depth of role engagement and psychological dimensions of volunteer involvement and their impact on health. The study focused on the impacts of volunteer hours, role engagement, and engagement profiles on the well-being and health of midlife and older adults. The research encompassed 665 participants aged 55 and above in Hong Kong. Initially, participants were classified into three categories based on volunteer hours (non-volunteers, 1-49 hours annually, 50 hours or more annually). Subsequently, they were segmented into four levels of role engagement (non-volunteers, low, medium, high) and six volunteering profiles (non-volunteers, occasional, regular, engaged, active, dedicated volunteers) considering volunteer hours and engagement levels. Results from multivariate regression analyses indicated that individuals volunteering over 50 hours annually exhibited enhanced subjective well-being (β = 0.18, p < 0.001) and self-rated health (β = 0.10, p < 0.01) compared to non-volunteers. Those with medium and high engagement levels also reported improved subjective well-being (Medium: β = 0.13, p < 0.001; High: β = 0.18, p < 0.001) and self-rated health (Medium: β = 0.08, p < 0.05; High: β = 0.07, p < 0.05) compared to non-volunteers. Cognitive benefits were consistent across volunteer hours and engagement levels, with active and dedicated volunteers experiencing the most significant advantages in well-being, health, and cognition. The study underscores the importance of role engagement in volunteering for late-life well-being and health, suggesting implications for integrating volunteering into social prescribing for healthy aging.

